# The Physiological Action and Therapeutic Use of Chloral

**Published:** 1871-08

**Authors:** 


*The Physiological action and Therapeutic Use of Chloral.* By J. B. Andrews, M. D.

Dr. Andrews presents in this pamphlet, which is a reprint from the American Journal of Insanity, the results of his experimental investigations of the effect of Chloral upon the circulation by means of the sphymograph, the tracings of which are illustrated. From these experiments, he concludes, in regard to the physiological action, "1st. That the effect of chloral is to reduce the number of pulsations. 2d. That the primary action is to increase the force of the heart's action and arterial tension. 3d. That in large doses, within safe limits, the pulsations are not reduced in number proportionately to the size of the dose; but the effect is more prolonged. That the secondary effect is to diminish the force of the heart's action and the arterial tension."

